# Relapsing Polychondritis With Severe Airway Involvement Mimicking Refractory Asthma: A Diagnostic Challenge

**DOI:** 10.7759/cureus.116342

**Published:** 2026-09-16

**Authors:** Sruthi Beladev, Manisha Dubey, Seeta Patel

**Affiliations:** 1 Respiratory Medicine, Queen Elizabeth the Queen Mother Hospital, Margate, GBR; 2 Internal Medicine, Queen Elizabeth the Queen Mother Hospital, Margate, GBR

**Keywords:** airway obstruction, autoimmune disease, corticosteroid therapy, cyclophosphamide, obstructive sleep apnoea, refractory asthma, relapsing polychondritis, respiratory failure, tracheal wall thickening, tracheobronchial stenosis

## Abstract

Relapsing polychondritis (RP) is a rare immune-mediated disorder that can cause severe tracheobronchial involvement and life-threatening airway compromise. We present a diagnostically challenging case of a 30-year-old non-binary patient whose progressive respiratory symptoms were initially attributed to severe refractory asthma. Despite repeated courses of systemic corticosteroids and escalation of asthma-directed therapy, they developed recurrent breathlessness, wheeze, and inspiratory stridor. Pulmonary function testing demonstrated severe fixed airflow obstruction with minimal bronchodilator reversibility and a flow-volume loop suggestive of central airway obstruction. Serial CT imaging subsequently demonstrated progressive smooth thickening and narrowing of the tracheobronchial tree, with relative sparing of the posterior membranous wall and no significant pulmonary parenchymal disease. These findings, together with the clinical course, exclusion of important alternative diagnoses, and multidisciplinary assessment by respiratory, rheumatology, radiology, and tertiary airway specialists, supported a clinicoradiological diagnosis of airway-dominant RP. Despite high-dose corticosteroids and cyclophosphamide followed by maintenance immunosuppression, the patient continued to experience severe airway compromise and recurrent respiratory symptoms. This case highlights the importance of considering RP in patients with treatment-refractory asthma-like symptoms, particularly when inspiratory stridor, fixed airflow obstruction, and progressive tracheobronchial abnormalities are present. Early recognition of central airway involvement and multidisciplinary assessment are essential to minimise irreversible airway damage.

## Introduction

Relapsing polychondritis (RP) is a rare systemic autoimmune disorder that primarily affects tissues rich in cartilage and proteoglycans. The condition is characterised by recurrent episodes of inflammation that can lead to progressive destruction of cartilage in affected areas, most commonly the ears, nose, trachea, and costal cartilage. Although the precise aetiology of RP remains uncertain, it is thought to result from an aberrant autoimmune response directed against cartilage tissues [1].

We report a rare and diagnostically challenging case of RP in a 30-year-old non-binary patient whose symptoms were initially attributed to severe refractory asthma over a prolonged period. The case demonstrates the importance of recognising atypical respiratory features and characteristic radiological findings suggestive of RP in patients with recurrent steroid-responsive airway disease.

Respiratory tract involvement occurs in approximately 30-60% of patients with RP and is associated with a more severe disease course. Unlike asthma, which typically causes variable airflow obstruction with at least partial reversibility, tracheobronchial involvement in RP may cause progressive, fixed central airway narrowing due to inflammation, cartilage thickening, and structural damage. Patients may present with persistent wheeze, inspiratory stridor, and progressive breathlessness despite conventional asthma treatment, and severe airway involvement can result in respiratory failure. Airway-predominant disease may occur with few or no classical systemic manifestations, making recognition particularly challenging. In patients with apparent refractory asthma, the presence of inspiratory stridor, fixed poorly reversible airflow obstruction, or characteristic tracheobronchial abnormalities on imaging should therefore prompt evaluation for central airway disease and consideration of RP.

In this case, the patient's disease was predominantly characterised by tracheobronchial involvement, with additional extra-airway symptoms that were reported not sufficient to establish definite cartilage involvement.

## Case presentation

A 30-year-old non-binary patient who uses they/them pronouns initially presented to Queen Elizabeth the Queen Mother Hospital in June 2023 with acute shortness of breath. At the time of presentation, they were undergoing outpatient investigation for possible asthma and were using a salbutamol inhaler. Prior to admission, they had completed a course of antibiotics and oral corticosteroids prescribed by their general practitioner. The patient reported smoking marijuana more than five times a day for approximately five years. They stopped smoking in 2023. The patient was 163 cm tall and weighed 108 kg, corresponding to a BMI of 40.15 kg/m², consistent with class III obesity.

On arrival to the emergency department, they were significantly breathless and unable to speak in full sentences. Clinical examination demonstrated widespread expiratory wheeze with notable inspiratory stridor. The presence of inspiratory stridor alongside expiratory wheeze was an important clinical clue to possible upper or central airway obstruction, although this was initially managed as an asthma exacerbation. Peak expiratory flow rate was 140 L/min. They were managed as an acute exacerbation of newly diagnosed asthma with oxygen therapy, nebulised bronchodilators, systemic corticosteroids, and inhaled maintenance therapy, including a beclomethasone/formoterol Maintenance and Reliever Therapy (MART) regimen alongside salbutamol.

The patient had no formally established diagnosis of asthma before this admission and had not undergone spirometry or formal pulmonary function testing. They reported multiple hospital admissions during early childhood, up to the age of five years, when they were treated for bronchiolitis and intermittently prescribed inhalers. They described exertional breathlessness during their school years but had remained relatively stable until contracting COVID-19 in January 2022, after which they developed progressive exertional dyspnoea.

Additional social history revealed a period of homelessness during the preceding two years, leading to concerns regarding possible exposure to infectious diseases such as tuberculosis. They were awaiting outpatient respiratory assessment at the time of their initial hospital admission. Given the patient's history of homelessness and the associated potential risk of tuberculosis exposure, infectious disease screening was performed. HIV and hepatitis B serology were negative, and QuantiFERON-TB testing for latent tuberculosis was also negative.

Pulmonary function testing performed in August 2023 demonstrated severe airflow obstruction. Pre-bronchodilator forced expiratory volume in one second (FEV1) was 1.05 L (33% predicted), increasing to 1.13 L (35% predicted) following 400 micrograms of inhaled salbutamol, representing an absolute increase of 0.08 L (80 mL; 7.6%). Forced vital capacity (FVC) increased from 3.90 L (106% predicted) to 4.09 L (111% predicted), while the FEV1/FVC ratio remained markedly reduced at 0.27 and 0.28, respectively. These findings did not demonstrate a significant bronchodilator response. The flow-volume loop was subsequently reviewed and raised concern for large/central airway obstruction. The physiologist noted difficulty performing a maximal forced expiratory manoeuvre because of artefact on the loop, and no greater values were achieved with pre- or post-bronchodilator slow vital capacity manoeuvres. The peak eosinophil count was 0.3 × 10⁹/L, which was within the laboratory reference range.

Following the initial presentation in June 2023, the patient continued to experience recurrent episodes of breathlessness, wheeze, cough, and chest tightness, which were initially attributed to asthma. Despite ongoing asthma-directed treatment, their respiratory symptoms persisted and progressively worsened, leading to recurrent hospital admissions from November 2024 onwards. They had six admissions between November 2024 and September 2025, during which they were repeatedly treated for presumed asthma exacerbations with systemic corticosteroids, nebulised bronchodilators, antibiotics, and escalation of inhaled therapies, including montelukast and tiotropium.

A CT of the thorax performed in November 2024 (Figures 1, 2) was initially reported as showing no significant pulmonary parenchymal abnormality or emphysema. Despite aggressive asthma-directed therapy, their symptoms continued to progress. Multiple emergency department visits occurred in July and August 2025 for persistent breathlessness and wheeze. CT pulmonary angiography performed in August 2025 (Figures 3, 4) demonstrated diffuse narrowing of the tracheobronchial tree despite otherwise clear lung fields and the absence of pulmonary embolism. In light of these findings, the November 2024 CT was retrospectively reviewed by specialist radiologists and was found to show smooth thickening of the tracheal cartilaginous walls with relative sparing of the posterior membranous wall and areas of airway narrowing, indicating that tracheobronchial abnormalities were already present.

**Figure 1:**
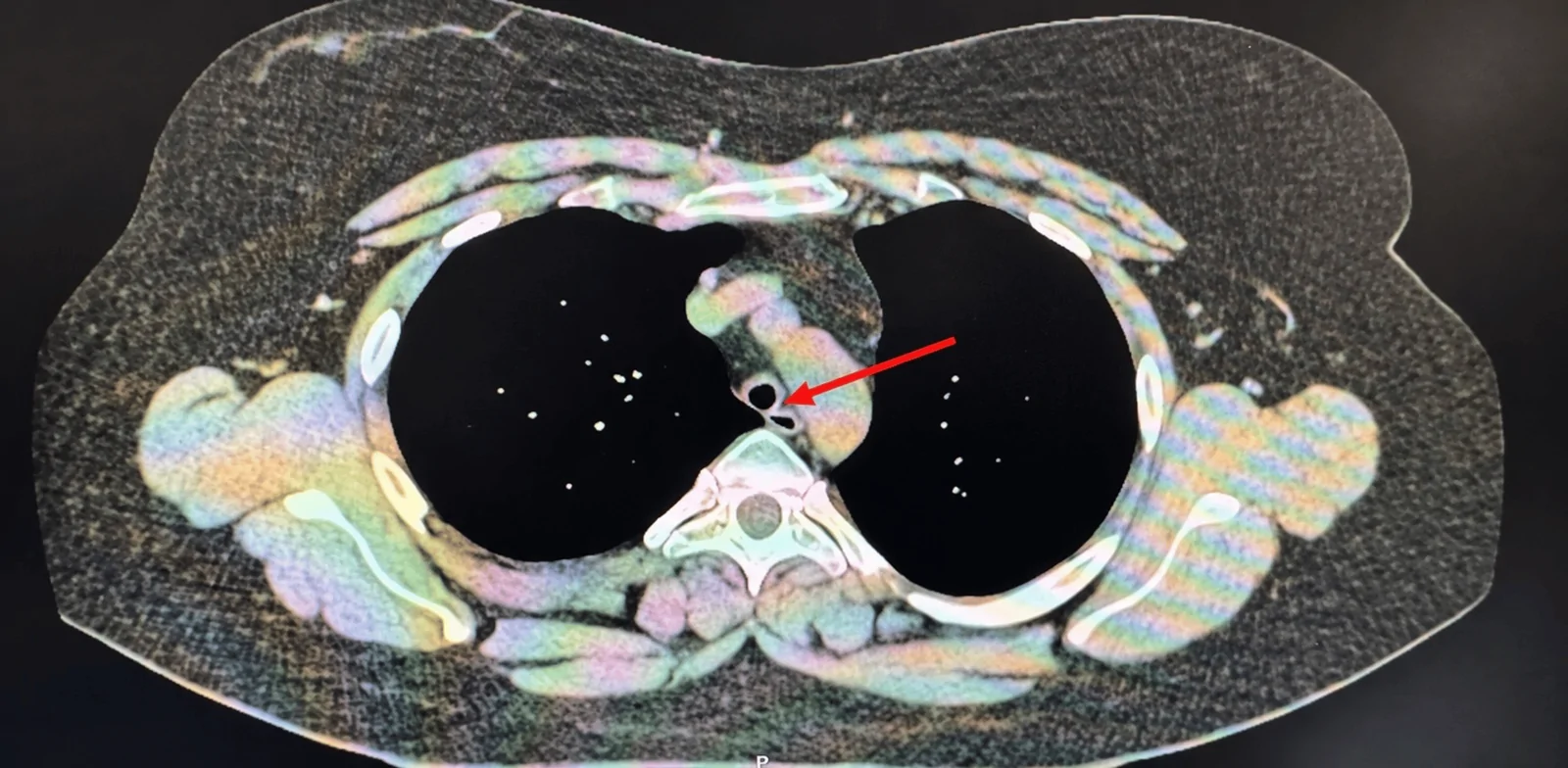
Axial contrast-enhanced CT of the thorax (November 2024) showing sparing of the posterior membranous wall with thickening of the anterior and lateral tracheal walls.

**Figure 2:**
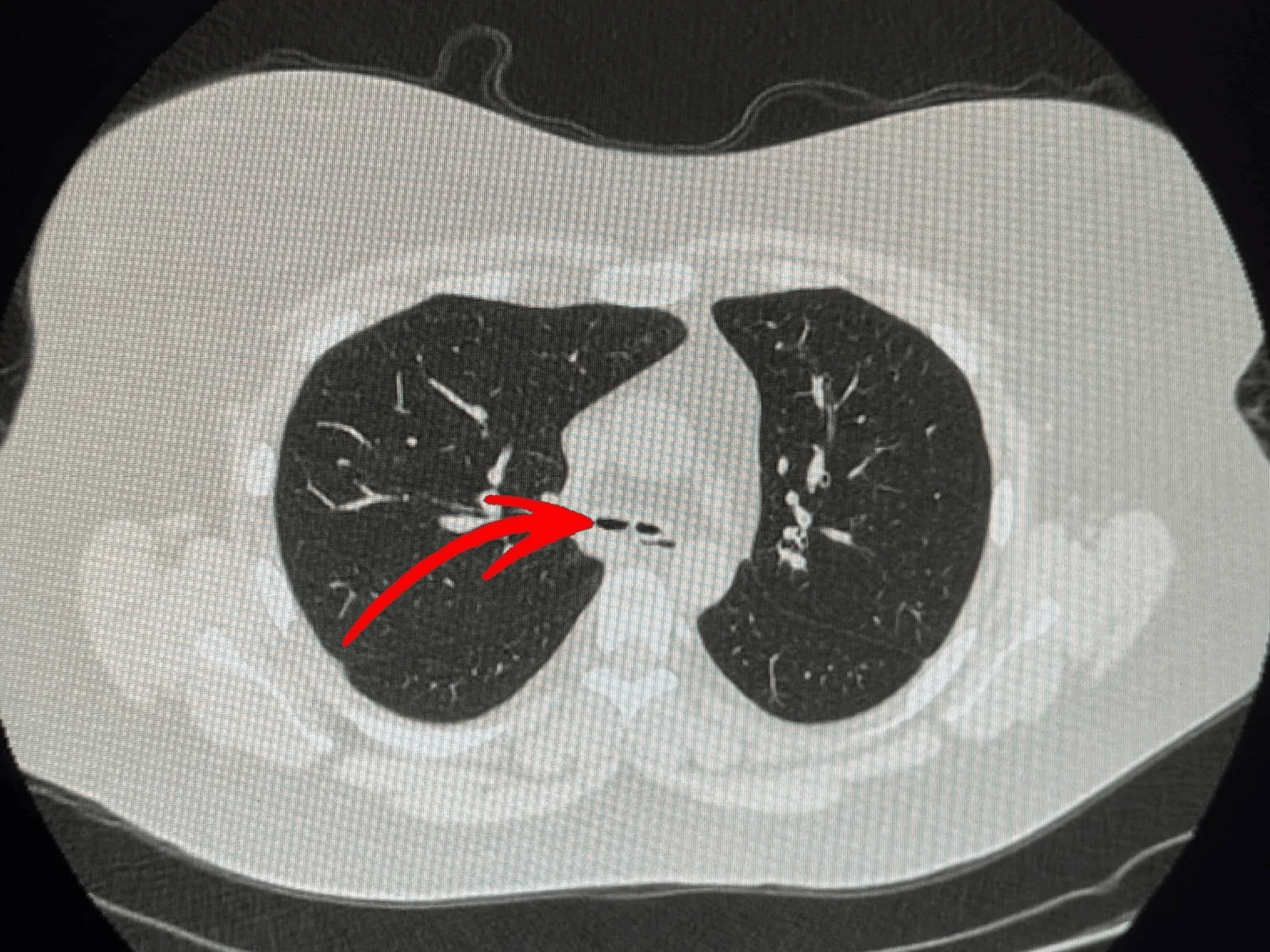
CT of the chest (November 2024) showing focal narrowing of the bronchial lumen, illustrating predominant respiratory tract involvement in relapsing polychondritis.

**Figure 3:**
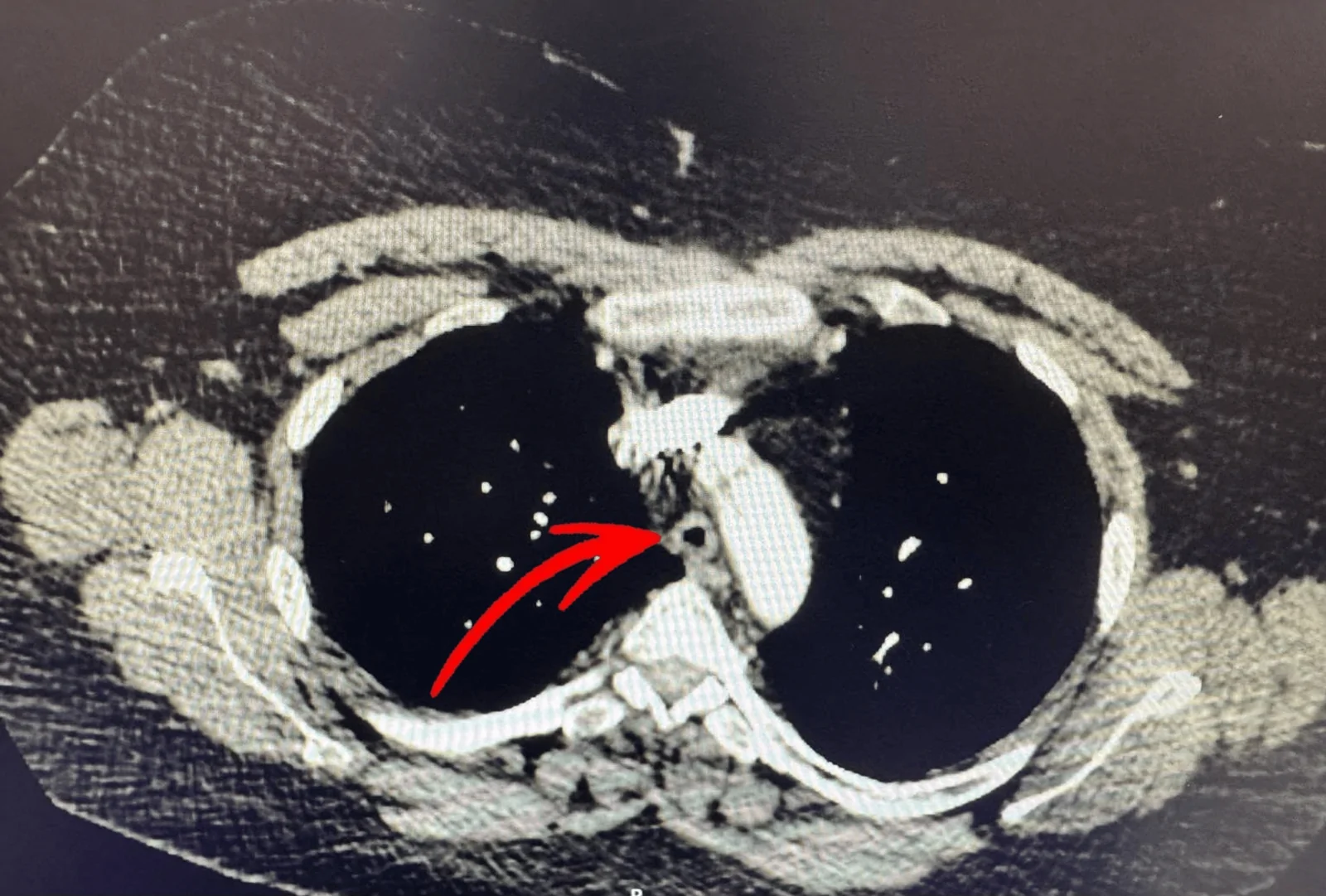
CT pulmonary angiogram (CTPA) obtained in August 2025 demonstrating diffuse narrowing of the tracheobronchial tree compared to the CT from November 2024.

**Figure 4:**
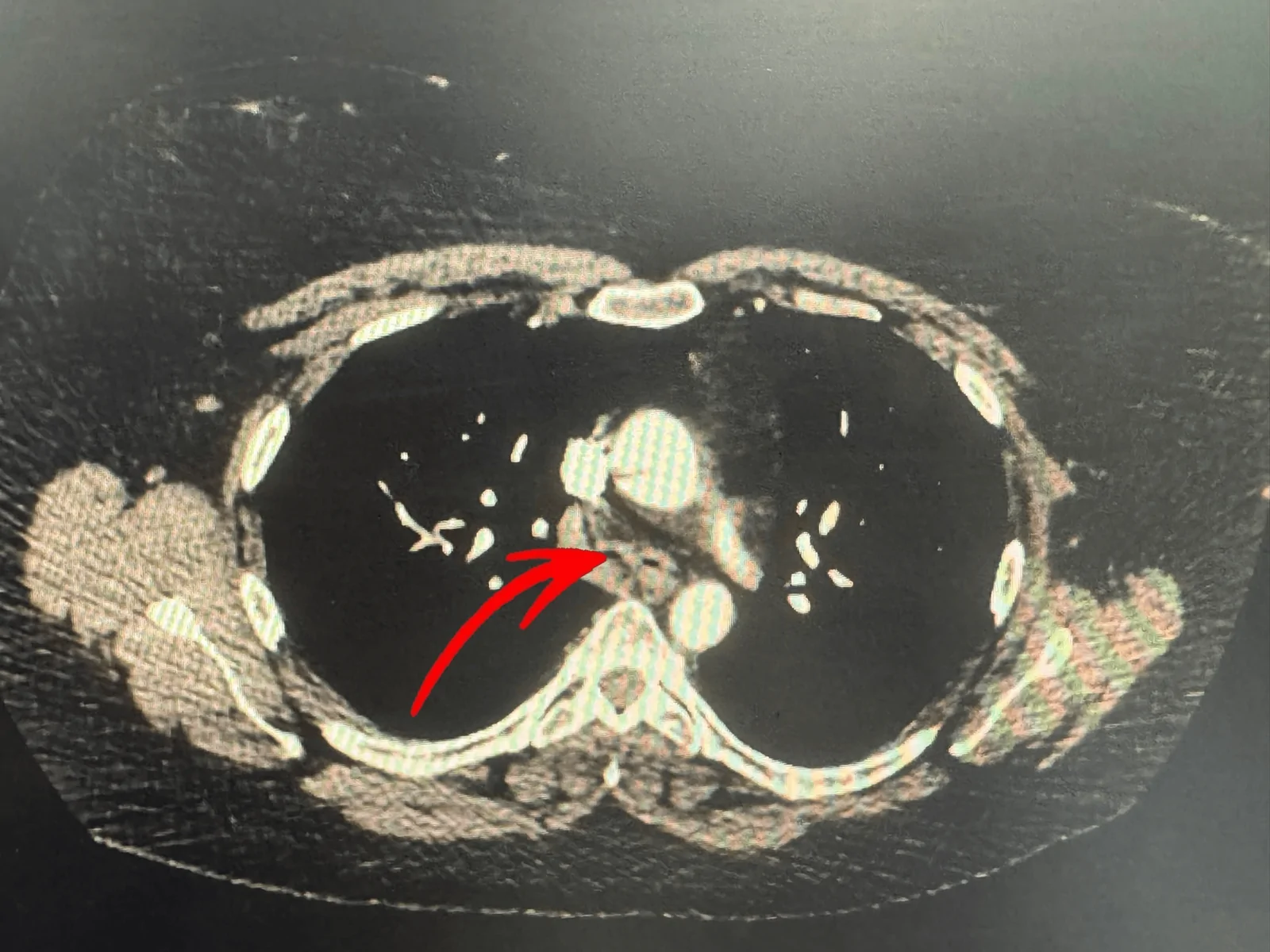
CTPA from August 2025 showing diffuse narrowing of bronchial lumen compared to CT from November 2024.

Sleep studies later confirmed obstructive sleep apnoea, and the patient awaited initiation of continuous positive airway pressure (CPAP) therapy. During admission in September 2025, further detailed history-taking revealed additional extra-respiratory symptoms. The patient reported intermittent redness of the earlobes, although there was no history or clinical evidence of typical auricular chondritis involving the cartilaginous pinna. They also reported recurrent nasal pain and chronic sinus discomfort, reduced hearing on the left side, and intermittent joint pains affecting the wrists and knees; however, these symptoms were not associated with objectively documented nasal chondritis, inflammatory arthritis, or active auricular inflammation. The reported hearing impairment was not objectively confirmed by formal audiological testing. Additional symptoms included occasional oral ulcers involving the palate. They denied inflammatory eye disease, haemoptysis, or skin rashes.

Laboratory investigations showed a C-reactive protein of 5 mg/L and an erythrocyte sedimentation rate of 2 mm/hour. Haemoglobin was 116 g/L, white cell count 11.0 × 10⁹/L, and platelet count 296 × 10⁹/L. Antinuclear antibody (ANA), antineutrophil cytoplasmic antibody (ANCA), and rheumatoid factor were negative. Complement levels were within normal limits, with C3 of 1.22 g/L and C4 of 0.20 g/L. Serum protein electrophoresis showed no paraprotein, and allergy testing was negative.

Clinical examination demonstrated bilateral ankle oedema and tenderness over the paranasal sinuses. There was no active auricular inflammation or synovitis at the time of review. The respiratory team reviewed the patient's repeated admissions and serial imaging, noting progressive tracheobronchial narrowing and the absence of significant pulmonary parenchymal disease. It also became apparent that, over the preceding year, they had received more than 12 courses of systemic corticosteroids for presumed asthma exacerbations, often with temporary symptomatic improvement. The combination of progressive tracheobronchial abnormalities, severe poorly reversible airflow obstruction, recurrent stridor, and persistent symptoms despite asthma-directed treatment raised strong suspicion for RP with tracheobronchial involvement. Discussions with the Royal Brompton airway team supported this diagnostic consideration. Recommendations included initiation of high-dose prednisolone with proton pump inhibitor therapy, *Pneumocystis jirovecii* pneumonia prophylaxis, bone protection therapy, and rheumatology involvement.

The local rheumatology team advised a comprehensive vasculitis screen, including ANCA, which was negative, and there was no clinical, biochemical, or radiological evidence of systemic vasculitis. CT sinus imaging did not demonstrate definitive upper airway features of RP. Nevertheless, the combination of progressive breathlessness, severe fixed airway obstruction, recurrent steroid responsiveness, and progressive tracheobronchial abnormalities strongly supported the diagnosis. The patient had experienced repeated courses of systemic corticosteroids for presumed asthma, including more than 12 courses during the year preceding diagnosis. Their weight increased from 108 kg in June 2023 to approximately 115 kg in June 2024 and 147.6 kg by September 2026. Although the cumulative corticosteroid exposure may have contributed to this weight gain, a direct causal relationship cannot be established. The subsequent severe obesity was associated with obstructive sleep apnoea and episodes of fluid overload requiring diuretic therapy.

A repeat CT of the neck with contrast in October 2025 compared with a CT dated May 2025 showed stable appearances with no salivary gland abnormality; however, the trachea was concentrically thickened and had a very small luminal calibre consistent with the history of RP.

The patient was commenced on cyclophosphamide therapy in addition to corticosteroids. They also received cotrimoxazole prophylaxis, calcium/vitamin D supplementation, proton pump inhibitor therapy, carbocisteine, montelukast, and ongoing CPAP therapy along with their tiotropium inhaler with AeroChamber, beclomethasone 200 microgram/formoterol 6 microgram inhaler, and salbutamol inhaler.

Despite initiation of immunosuppressive therapy, the patient continued to experience recurrent admissions throughout late 2025 and early 2026 with worsening shortness of breath, cough, peripheral oedema, and fluid overload. ENT specialists concluded that tracheostomy would not provide benefit because the critical narrowing was located distal to the site accessible by conventional tracheostomy.

CT imaging in January 2026 demonstrated narrowing of the trachea and proximal bronchi (Figure 5). The radiology report noted that the trachea was more significantly narrowed in the transverse than in the anteroposterior dimension, measuring approximately 2 cm transversely at some points, while the proximal bronchi appeared narrowed in the anteroposterior dimension, measuring approximately 1-2 mm at some points. As this was a single-phase CT examination, the report noted that the degree of airway narrowing should be interpreted in this context. Earlier CT imaging had demonstrated smooth thickening of the tracheal cartilaginous walls with relative sparing of the posterior membranous wall. Taken together with the progressive clinical course and previous imaging findings, these appearances supported severe tracheobronchial involvement in the context of the multidisciplinary diagnosis of RP. Images were reviewed jointly with tertiary airway specialists during multidisciplinary meetings.

**Figure 5:**
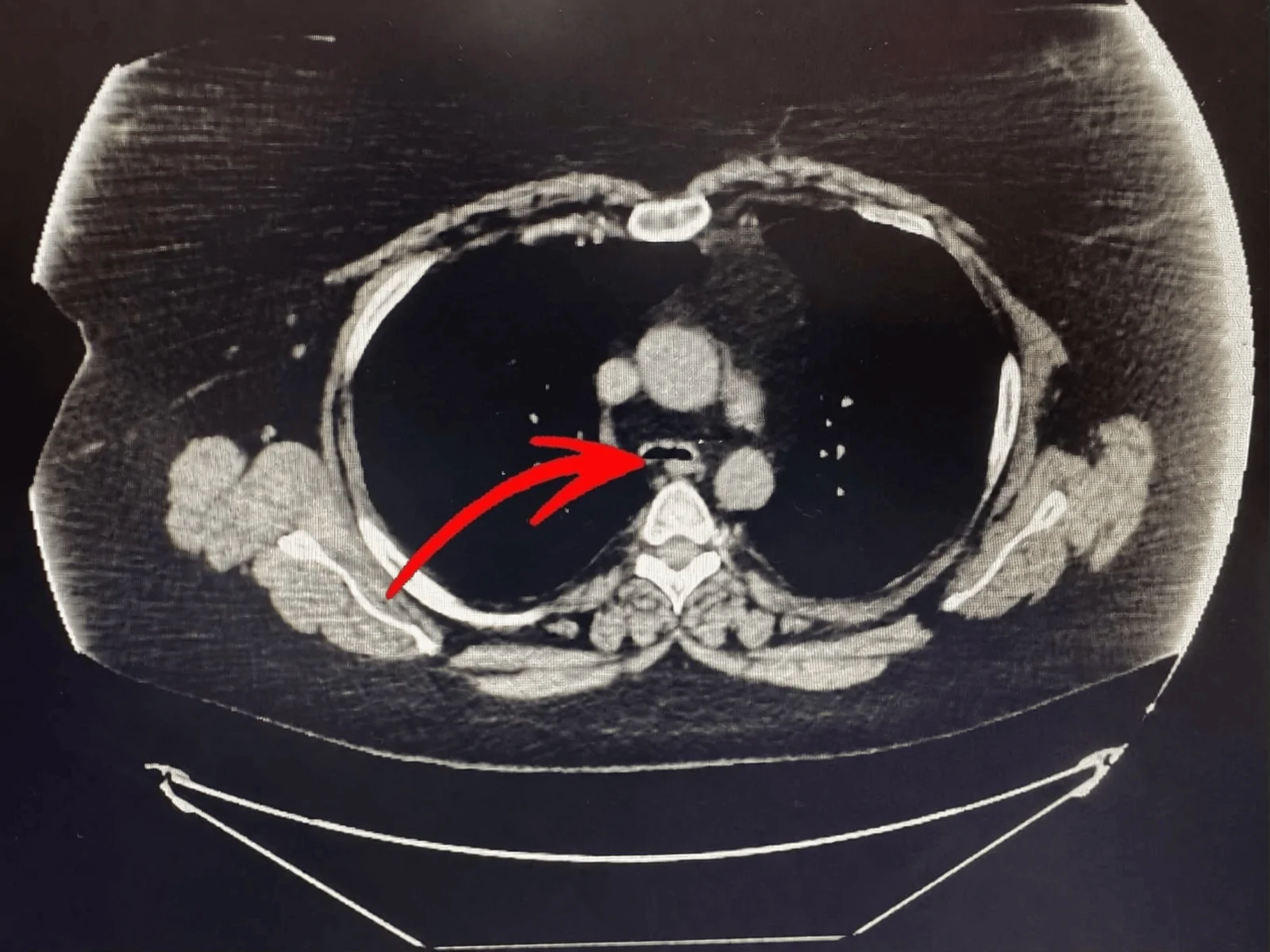
Axial CT of the chest from January 2026 showing marked narrowing to the tracheal lumen in keeping with airway involvement in relapsing polychondritis.

The patient ultimately received 10 cycles of intravenous cyclophosphamide 1,200 mg because of the severity and progressive nature of the airway disease. Following completion of cyclophosphamide, they were commenced on methotrexate 20 mg once weekly and hydroxychloroquine 200 mg once daily following multidisciplinary discussion between the respiratory and rheumatology teams. Prednisolone was continued at 40 mg once daily, with a plan for gradual tapering according to clinical response and specialist review. The ongoing immunosuppressive regimen was determined according to the patient's clinical course and multidisciplinary assessment, with close follow-up by local respiratory, rheumatology, and tertiary airway and rheumatology teams.

They continue to require home CPAP therapy and prolonged corticosteroid treatment with gradual tapering attempts. Emergency airway planning recommendations advised use of a size 7 endotracheal tube with positive pressure ventilation in the event of acute deterioration, acknowledging anticipated difficult front-of-neck airway access.

Despite initiation of immunosuppressive therapy, the patient continued to experience recurrent respiratory admissions and persistent severe airway compromise, with ongoing respiratory symptoms and continued requirement for corticosteroid therapy. These findings indicated persistent severe respiratory disease despite treatment. Given the persistent severe airway disease despite corticosteroids and cyclophosphamide, biologic therapy is being considered. Anti-TNF therapy or tocilizumab are potential options based on published experience in severe respiratory RP, with the final choice dependent on the patient's clinical phenotype, previous treatment response, safety considerations, and multidisciplinary specialist assessment. No biologic therapy has been commenced to date.

Given the progressive and life-threatening nature of the airway disease, persistent respiratory symptoms despite immunosuppression, and the recognised adverse prognosis associated with severe respiratory involvement, palliative care was involved alongside active specialist treatment. This input focused on symptom control, psychosocial support, and advance care planning rather than representing a transition away from disease-directed treatment.

To provide a clear overview of the patient's clinical course, including the progression of respiratory symptoms, key investigations, treatment interventions, and subsequent diagnosis, a clinical timeline is presented in Table 1.

**Table 1:** Clinical timeline of the patient’s presentation, investigations, and management. CTPA: CT pulmonary angiogram; CPAP: continuous positive airway pressure.

Date/period	Clinical course/investigation	Key findings/management
January 2022	COVID-19 infection	Progressive exertional dyspnoea developed following infection
June 2023	Initial hospital presentation	Acute severe breathlessness, expiratory wheeze and inspiratory stridor; initially treated as asthma exacerbation
August 2023	Pulmonary function testing	Severe fixed airflow obstruction: FEV1 1.05 L (33%) pre-bronchodilator and 1.13 L (35%) post-bronchodilator, with minimal bronchodilator response; flow-volume loop raised concern for central airway obstruction
November 2024	CT thorax	Initially reported as without significant pulmonary abnormality; retrospective specialist review later demonstrated tracheal cartilage thickening and airway narrowing
November 2024 to September 2025	Recurrent admissions	Six admissions for worsening dyspnoea, wheeze, and cough; repeatedly treated as asthma exacerbations with systemic corticosteroids, nebulised bronchodilators, antibiotics, and escalation of inhaled therapy
August 2025	CTPA	Diffuse narrowing of the tracheobronchial tree with clear lung fields and no pulmonary embolism
August to September 2025	Specialist radiology review and further clinical assessment	A retrospective review of previous CT demonstrated smooth tracheal cartilage thickening with relative sparing of the posterior membranous wall; further history revealed intermittent auricular symptoms, nasal symptoms, hearing impairment and arthralgia
September 2025	Multidisciplinary assessment	Airway-dominant relapsing polychondritis suspected; rheumatology and tertiary airway teams involved
October 2025	CT neck with contrast	Concentric tracheal thickening with markedly reduced luminal calibre; no significant salivary gland abnormality
Late 2025 to early 2026	Progressive airway disease	Recurrent respiratory admissions despite immunosuppressive treatment; persistent severe airway narrowing and respiratory compromise
January 2026	CT chest	Marked narrowing of the trachea and proximal bronchi, with severe luminal reduction
May 2026	Completion of cyclophosphamide	Ten cycles of intravenous cyclophosphamide completed; subsequently commenced on methotrexate and hydroxychloroquine
2026	Ongoing management	Persistent severe airway compromise and steroid dependence; continued CPAP and multidisciplinary respiratory, rheumatology and tertiary airway follow-up; biologic therapy under consideration

## Discussion

RP is an uncommon immune-mediated disorder characterised by recurrent inflammation and progressive destruction of cartilaginous structures. Auricular chondritis is the most characteristic clinical manifestation, while approximately 30% of cases are associated with other systemic conditions, particularly systemic vasculitis and myelodysplastic syndromes [2]. In contrast to the typical phenotype, our patient had predominantly tracheobronchial involvement, resulting in severe fixed airflow obstruction and recurrent treatment for presumed refractory asthma before the diagnosis of RP was established.

Epidemiology

RP is a rare disorder, with disease onset most commonly occurring between 40 and 60 years of age [2]. Our patient developed severe airway-dominant disease at a younger age than typically reported, with progressive respiratory symptoms becoming the predominant manifestation during early adulthood. This atypical age at presentation, together with the absence of prominent classical systemic features, contributed to the diagnostic challenge.

Pathogenesis

The aetiopathogenesis of RP remains incompletely understood and is thought to involve genetic susceptibility, environmental factors, and autoimmune mechanisms directed against cartilage-associated components [3]. Proposed serological markers, including antibodies to type II collagen and matrilin-1, lack sufficient sensitivity and specificity for routine diagnosis [3]. In this case, the patient reported worsening respiratory symptoms following COVID-19 infection in January 2022 and vaccination later that year; however, these represent temporal associations only, and no causal relationship with the development or progression of RP can be established.

Clinical manifestations

Tracheobronchial involvement is an important manifestation of RP, occurring in approximately 60% of patients. It may present with cough, progressive dyspnoea, wheeze, or stridor and can cause progressive airway narrowing, fixed stenosis, tracheobronchomalacia, and bronchiectasis. These respiratory features may closely mimic severe or refractory asthma, particularly when classical systemic manifestations are absent. Other recognised manifestations of RP include auricular, nasal, articular, ocular, audiovestibular, cutaneous, cardiovascular, and neurological involvement [4].

In our patient, airway disease was the predominant manifestation and preceded recognition of RP by several years. They were repeatedly treated for presumed severe asthma despite progressive fixed airflow obstruction and recurrent stridor. Although intermittent earlobe erythema, nasal symptoms, and arthralgia were reported, there was no objectively documented auricular chondritis, inflammatory arthritis, ocular, cardiovascular, neurological, or cutaneous involvement. The predominantly airway-dominant presentation therefore contributed substantially to the diagnostic uncertainty. Persistent wheeze, stridor, and poorly reversible airflow obstruction should prompt consideration of central airway disease and alternative diagnoses such as RP when symptoms remain refractory to conventional asthma treatment. Table 2 presents the diagnostic criteria for relapsing polychondritis.

**Table 2:** Diagnostic criteria for relapsing polychondritis [8].

Criteria set	Clinical features/criteria	Diagnostic requirement
McAdam et al. (1976) [5]	Bilateral auricular chondritis, nasal chondritis, respiratory tract chondritis, non-erosive seronegative polyarthritis, ocular inflammation, and audiovestibular damage	≥3 criteria
Damiani et al. (1979) [6]	A criteria: Bilateral auricular chondritis, nasal cartilage inflammation, respiratory tract chondritis, non-erosive seronegative polyarthritis, ocular inflammation, and audiovestibular damage; B criterion: Histological confirmation; C criterion: Positive response to corticosteroids or dapsone	≥3 A criteria; ≥1 A + B criterion; ≥2 A criteria + C criterion
Michet et al. (1986) [7]	A criteria: Auricular cartilage inflammation, nasal cartilage inflammation, and laryngotracheal cartilage inflammation; B criteria: Seronegative arthritis, ocular inflammation, hearing loss, and vestibular dysfunction	≥2 A criteria; ≥1 A + 2 B criteria

In this case, the diagnosis was supported by severe laryngotracheobronchial involvement demonstrated on serial CT imaging, together with a history of nasal symptoms, intermittent ear symptoms, hearing impairment, and arthralgia. However, the patient did not have objectively documented bilateral auricular chondritis, definite nasal chondritis, non-erosive seronegative polyarthritis, ocular inflammation, or vestibular dysfunction and therefore did not fulfil the required number of clinical criteria for the McAdam, Damiani, and Levine, or Michet classification criteria sets. Although respiratory tract chondritis was clearly demonstrated, the additional reported symptoms were not considered sufficient to establish the required criteria because they lacked objective clinical confirmation. Cartilage biopsy was not performed, and therefore, histological confirmation was unavailable. The diagnosis was consequently based on the overall clinicoradiological presentation and multidisciplinary specialist assessment, supported particularly by the characteristic smooth tracheobronchial cartilage thickening with relative sparing of the posterior membranous wall, progressive fixed airflow obstruction, recurrent stridor, and exclusion of major alternative diagnoses. The absence of histological confirmation and incomplete fulfilment of established classification criteria represent important limitations of the diagnostic certainty in this case.

Laboratory tests

No laboratory marker is specific for the diagnosis of RP. Inflammatory markers may be elevated during active disease, while autoantibodies including ANA and ANCA may be detected, particularly in patients with associated autoimmune disease or vasculitis [9].

In our patient, routine haematological and inflammatory parameters were generally unremarkable, and the immunological, electrophoretic, and allergy investigations did not identify an alternative systemic autoimmune, vasculitic, or allergic/eosinophilic disorder. These findings did not establish the diagnosis of RP, but importantly, the absence of elevated inflammatory markers did not exclude the disease, particularly given its predominantly airway-limited phenotype. The diagnosis was ultimately supported by the clinical presentation, progressive fixed airflow obstruction, and characteristic tracheobronchial imaging findings.

Pulmonary function tests

Pulmonary function testing, including spirometry and flow-volume loops, can provide important physiological evidence of central airway obstruction in RP [9]. In retrospect, the severe, poorly reversible airflow obstruction and flow-volume loop findings identified on pulmonary function testing in August 2023 represented an important early physiological clue to central airway disease. This was subsequently supported by serial CT imaging demonstrating progressive, diffuse tracheobronchial narrowing with characteristic thickening of the cartilaginous airway walls and relative sparing of the posterior membranous wall, consistent with airway involvement in RP.

Bronchoscopy

Bronchoscopy can provide direct assessment of airway inflammation, stenosis, and tracheobronchomalacia in RP and may assist in defining the extent of airway involvement [10]. However, in patients with severe airway narrowing, bronchoscopy may carry a significant risk of respiratory compromise, including airway oedema and acute deterioration [10]. In our patient, bronchoscopy was not performed because of the severity of the fixed airway narrowing and the perceived risk of procedural respiratory compromise. Consequently, cross-sectional imaging was used to characterise the extent and pattern of tracheobronchial involvement.

Imaging

CT imaging is an important tool for identifying airway involvement in RP. Typical findings include tracheobronchial wall thickening and luminal narrowing, subglottic stenosis, cartilage calcification, bronchiectasis, and airway collapse. Smooth thickening of the tracheobronchial walls with luminal narrowing is particularly characteristic [11]. 18F-FDG PET/CT may provide additional information regarding inflammatory activity and the distribution of cartilaginous involvement but was not required in our patient [12].

In our patient, serial CT imaging demonstrated smooth thickening of the tracheal cartilage with progressive tracheobronchial narrowing and relative sparing of the posterior membranous wall, without significant pulmonary parenchymal disease or emphysema. These findings, together with progressive fixed airflow obstruction, recurrent stridor, and treatment-refractory asthma-like symptoms, provided the key objective evidence supporting the diagnosis of airway-dominant RP [11].

Conditions associated with RP

Vasculitis is a recognised systemic association of RP and may contribute to morbidity and prognosis [13]. In our patient, vasculitis was considered because of the respiratory and upper airway manifestations; however, extensive evaluation, including ANA and ANCA serology, demonstrated no clinical, biochemical, or radiological evidence of systemic vasculitis or other associated autoimmune disease. The absence of associated systemic disease further supports the predominantly airway-dominant phenotype observed in this case.

Differential diagnosis

The diagnosis of RP is primarily clinical, supported by characteristic imaging findings and, where appropriate, histological assessment. In patients presenting with persistent wheeze, stridor, or fixed airflow obstruction, important differential diagnoses include severe asthma, granulomatosis with polyangiitis, sarcoidosis, tracheobronchopathia osteochondroplastica, amyloidosis, and other inflammatory or infiltrative disorders affecting the central airway [14].

In this patient, several important differential diagnoses were considered. Severe asthma was initially the working diagnosis; however, the persistent fixed airflow obstruction, minimal bronchodilator response, inspiratory stridor, progressive central airway narrowing, and characteristic CT appearance with smooth tracheobronchial cartilage thickening and relative sparing of the posterior membranous wall were atypical for asthma. Granulomatosis with polyangiitis and other systemic vasculitides were considered because of the upper airway and respiratory manifestations, but ANCA testing was negative and there was no clinical, biochemical, or radiological evidence of systemic vasculitis. Infectious causes, including tuberculosis, were also considered given the history of homelessness; HIV, hepatitis B, and latent tuberculosis screening with QuantiFERON-TB were negative, and there was no supporting clinical or radiological evidence of active tuberculosis. Sarcoidosis was considered but was not supported by the absence of pulmonary parenchymal disease or significant lymphadenopathy. The absence of a pulmonary mass, endoluminal lesion, or significant lymphadenopathy on serial imaging also made an infiltrative or malignant airway process less likely. Overall, the progressive airway narrowing and characteristic CT pattern, together with the clinical features and exclusion of major alternative diagnoses, supported a clinicoradiological diagnosis of airway-dominant RP. IgG4-related disease was also considered as a potential cause of tracheobronchial wall thickening; however, the clinical phenotype and characteristic smooth, cartilage-predominant thickening with relative sparing of the posterior membranous wall were more supportive of RP.

Serological investigations are non-specific in RP, and currently proposed biomarkers do not have sufficient diagnostic accuracy for routine clinical use. Cartilage biopsy may provide supportive evidence in selected cases but is not routinely required when the clinical and radiological findings are characteristic [14].

Management of RP

Management of RP is guided by disease severity and organ involvement. Systemic corticosteroids are commonly used for significant disease, with conventional immunosuppressive and biologic therapies considered in severe or refractory cases, particularly with airway involvement [15,16]. Airway intervention, including stenting, may be required in selected patients with severe structural stenosis, although complications limit its use [17]. Respiratory involvement is an important contributor to morbidity in RP, with tracheomalacia and respiratory dysfunction reported in affected patients [18].

In our patient, the severity and progressive nature of the airway disease prompted treatment with high-dose corticosteroids and cyclophosphamide, followed by methotrexate and hydroxychloroquine as maintenance therapy. Despite immunosuppression, they remained steroid-dependent with persistent severe airway compromise and recurrent respiratory symptoms. Biologic therapy, including anti-TNF treatment or tocilizumab, is being considered because of refractory disease. Airway stenting was not pursued because of the diffuse and distal nature of the tracheobronchial narrowing. Ongoing management involves respiratory, rheumatology, and tertiary airway teams, with palliative care input for symptom management and advance care planning.

Prognosis and survival of RP

RP is associated with increased mortality compared with the general population. In a cohort of 106 patients from King’s College London and Guy’s and St Thomas’ NHS Foundation Trust, mortality was more than twice that expected in the general population, with a standardised mortality ratio of 2.16 [19]. Severe respiratory involvement, particularly laryngotracheal disease, is an important contributor to morbidity and adverse outcomes [19].

In our patient, severe progressive laryngotracheobronchial disease resulted in fixed airflow obstruction, recurrent respiratory admissions, and significant respiratory disability despite relatively limited systemic involvement. This case highlights that severe airway-dominant disease can result in substantial morbidity and poor clinical outcome, emphasising the importance of early recognition of airway involvement in patients with persistent or treatment-refractory asthma-like symptoms.

Limitations

This case has several limitations. The patient did not clearly fulfil a single established diagnostic criteria set for RP, as the respiratory involvement was objectively demonstrated, but the additional required clinical manifestations were not sufficiently established, and histological confirmation was not obtained. Several extra-respiratory features were based predominantly on patient-reported symptoms rather than objectively documented chondritis. Although serial CT imaging demonstrated severe fixed tracheobronchial narrowing with characteristic cartilage-predominant wall thickening and relative sparing of the posterior membranous wall, bronchoscopy and formal dynamic expiratory airway assessment were not performed because of the severity of the airway disease and the perceived risk of respiratory compromise. Therefore, possible coexisting dynamic airway collapse or tracheobronchomalacia could not be definitively assessed. The diagnosis was consequently based on the overall clinicoradiological presentation, exclusion of major alternative diagnoses, and multidisciplinary assessment by respiratory, rheumatology, radiology, and tertiary airway specialists. Finally, the reported worsening of respiratory symptoms following COVID-19 infection and vaccination represents a temporal association only, and no causal relationship can be established from this case.

## Conclusions

This case highlights the importance of considering central airway disease in patients treated as having refractory asthma who develop inspiratory stridor, fixed airflow obstruction, and minimal bronchodilator reversibility. In this patient, progressive tracheobronchial narrowing with characteristic cartilage-predominant wall thickening on serial CT imaging, together with the clinical presentation, supported a clinicoradiological diagnosis of airway-dominant RP following multidisciplinary assessment. Although histological confirmation was not obtained and established classification criteria were not fully met, early recognition of characteristic airway features is important to facilitate appropriate multidisciplinary evaluation and management before irreversible airway damage develops.
